# Causal associations between human gut microbiota and osteomyelitis: a Mendelian randomization study

**DOI:** 10.3389/fcimb.2024.1338989

**Published:** 2024-04-09

**Authors:** Wenxing Zeng, Yuheng Wu, Xiaoye Liang, Dejun Cun, Luyao Ma, Jingtao Zhang, Feng Huang, Ziwei Jiang

**Affiliations:** ^1^ First Clinical Medical College of Guangzhou University of Chinese Medicine, Guangzhou, China; ^2^ Seventh Clinical Medical College of Guangzhou University of Chinese Medicine, Shenzhen, China; ^3^ Department of Traumatology and Orthopedics, The First Affiliated Hospital of Guangzhou University of Chinese Medicine, Guangzhou, China

**Keywords:** osteomyelitis, gut microbiota, Mendelian randomization, causal effect, genetic association

## Abstract

**Background:**

Recent studies have emphasized the role of gut microbiota in the onset and progression of osteomyelitis. However, the exact types of gut microbiota and their mechanisms of action remain unclear. Additionally, there is a lack of theoretical support for treatments that improve osteomyelitis by altering the gut microbiota.

**Methods:**

In our study, we utilized the largest genome-wide association study (GWAS) meta-analysis to date from the MiBioGen consortium, involving 13,400 participants. The GWAS data for osteomyelitis were sourced from the UK Biobank, which included 4,836 osteomyelitis cases and 486,484 controls. We employed a two-sample Mendelian randomization framework for a detailed investigation into the causal relationship between gut microbiota and osteomyelitis. Our methods included inverse variance weighting, MR-Egger, weighted median, and weighted mode approaches. Additionally, we applied Cochran’s Q statistic to assess the heterogeneity of the instrumental variable.

**Results:**

At the class level, Bacilli and Bacteroidia were positively correlated with the risk of osteomyelitis. At the order level, only *Bacteroidales* showed a positive association with osteomyelitis. At the genus level, an increased abundance of *Butyricimonas*, *Coprococcus3*, and *Tyzzerella3* was positively associated with the risk of osteomyelitis, whereas *Lachnospira* was negatively associated. Sensitivity analyses showed no evidence of heterogeneity or pleiotropy.

**Conclusion:**

This study reveals that classes Bacilli and Bacteroidia, order Bacteroidales, and genera *Butyricimonas*, *Coprococcus3*, and *Tyzzerella3* are implicated in increasing the risk of osteomyelitis, while the genus *Lachnospira* is associated with a reduced risk. Future investigations are warranted to elucidate the precise mechanisms through which these specific bacterial groups influence the pathophysiology of osteomyelitis

## 1 Introduction

Osteomyelitis (OM) an inflammatory bone disease primarily caused by microbial infections that lead to bone destruction, affects various skeletal areas, including the bone marrow, cortex, periosteum, and surrounding soft tissues (Lew and Waldvogel, 2004). In the United States, the incidence of OM has been rising, with cases increasing from 11.4 per 100,000 person-years between 1969 and 1979 to 24.4 per 100,000 person-years between 2000 and 2009. This trend is notably more pronounced in males and older age groups (Kremers et al., 2015). This increase also reflects the significant impact of environmental factors on OM risk. OM is typically caused by infections with various microorganisms, but in special cases like chronic non-bacterial OM, it manifests as an autoinflammatory bone disorder, ruling out infection as a cause. The pathogenesis of this condition is not well understood, yet some studies have pointed to strong environmental and genetic correlations (Hedrich et al., 2013).

The human body’s largest known commensal microbial community, the gut microbiota, consists of bacteria, fungi, viruses, and protozoa (Shkoporov and Hill, 2019). This community includes roughly 4 trillion microorganisms (Sender et al., 2016) and about 150,000 microbial genomes (Pasolli et al., 2019). The gut microbiota plays a crucial role in human metabolism, immune regulation, and maintaining the stability of the intestinal mucosal barrier (Hitch et al., 2022). Recent advances in microbiome research have led to a new understanding: the host microbiota can either maintain dynamic homeostasis with the host organism or exacerbate infectious states. This insight has sparked the hypothesis that the gut microbiota may significantly influence various infectious diseases, including OM (Chen et al., 2021). Recent developments in high-throughput sequencing technologies have enhanced our understanding of the relationship between gut microbiota and OM. For example, an animal study showed that altering the gut microbiota diversity in rats with oligofructose significantly boosted the population of the anti-inflammatory bacterium Bifidobacterium pseudolongum, thereby markedly reducing the severity of OM by impeding hyperinflammation onset and progression (Bui et al., 2022). Furthermore, several clinical studies have suggested that antibiotic usage can disrupt gut microbiota, potentially increasing the risk of bloodstream infections and severe health conditions (Ubeda et al., 2010; Ayres et al., 2012). However, most of the current research is observational, often constrained by small sample sizes and susceptible to confounding factors. While these studies suggest a link between gut microbiota and OM, they stop short of establishing a direct causal relationship.

Currently, a substantial body of research supports the extensive role of gut microbiota in human diseases. It is increasingly clear that the gut microbiota, along with specific microbial metabolites, impacts not only local processes such as the host’s inflammatory responses, nutrient absorption, and intestinal barrier function, but also broader systems. These include the immune system, glucose homeostasis, lipid metabolism, energy balance, nonalcoholic fatty liver disease, obesity, associated comorbidities, and other metabolic disorders. This is particularly relevant for autoinflammatory diseases like asthma, arthritis, colitis, diabetes, and lupus (Lo et al., 1991; Bach, 2002; Bodkhe et al., 2019; Marietta et al., 2019). Consequently, strategies focusing on the regulation of gut microbiota could offer new perspectives and approaches for treating autoimmune diseases. Prior studies have also established a connection between gut microbiota and the development of OM (Phillips et al., 2016).

Mendelian randomization (MR) utilizes single nucleotide polymorphisms (SNPs) as instrumental variables to investigate causal relationships between exposures and outcomes (Emdin et al., 2017). Unlike observational studies, MR leverages the random assortment of alleles during inheritance to bypass confounders and reverse causation, thus resembling the design of randomized controlled trials (Emdin et al., 2017; Bowden and Holmes, 2019). While MR has been employed in various studies to examine potential causal links between gut microbiota and different diseases (Xu et al., 2021; Chen et al., 2022; Lee, 2022) its application to explore possible causal connections between gut microbiota and OM risk remains unexplored.

In this study, we used Mendelian randomization analysis to comprehensively examine the potential causal relationship between gut microbiota and OM, with the goal of identifying specific bacterial taxa that might contribute to pathogenesis or offer protection.

## 2 Materials and methods

### 2.1 Study design

**Figure 1:**
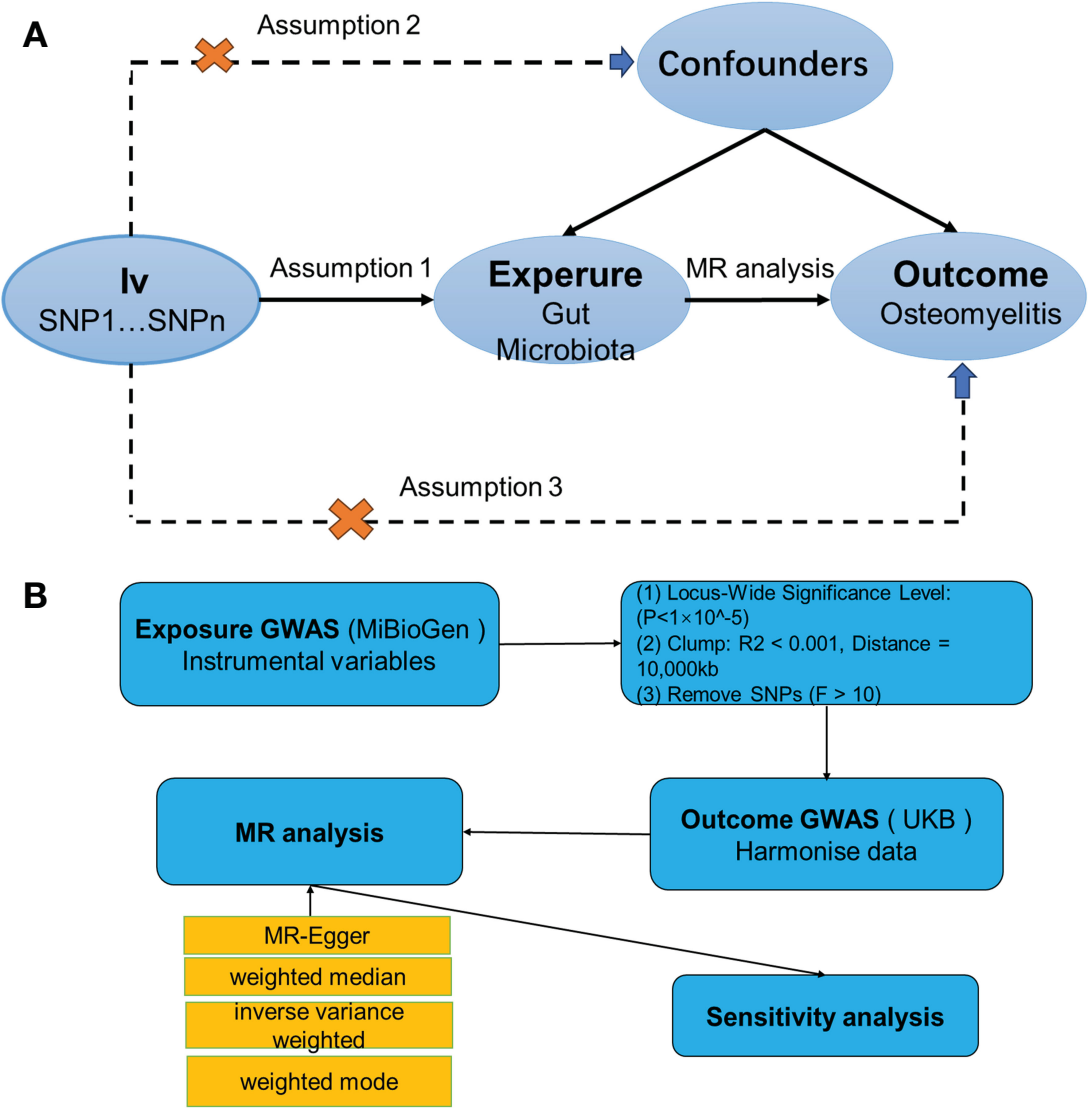
**(A)** Three assumptions of Mendelian randomization. **(B)** Flowchart of this Mendelian randomization study. GWAS, Genome Wide Association Studies; IV, Instrumental variable; SNP, single nucleotide polymorphism; MR, Mendelian randomization; IVW, Inverse-variance weighted; WM, Weighted median; UKB, UK Biobank.

MR is a methodological tool in human genetics that utilizes the random distribution properties of genetic variants during gamete formation and fertilization to infer causality (Davey Smith and Ebrahim, 2003). Its primary aim is to assess the causal relationship between exposures and outcomes empirically observed, thereby bypassing the confounding biases inherent in traditional epidemiological studies. To derive reliable results, as depicted in **Figure 1A**, a two-sample MR must meet three key assumptions (Bowden and Holmes, 2019): (1) the chosen SNPs show significant associations with gut microbiota; (2) these SNPs function independently of any potential confounders between the exposure and the outcome; and (3) the SNPs are associated with the outcome (OM) exclusively through their relationship with gut microbiota, eliminating any direct associations.

### 2.2 Data selection

#### 2.2.1 Exposure genome-wide association study: gut microbiota

The exposure data for this study came from a large-scale, multi-ethnic, genome-wide meta-analysis focusing on the associations between human genetic autosomal variants and gut microbiota, conducted by the MiBioGen Consortium (van der Velde et al., 2019). This analysis gathered 16S rRNA gene sequencing profiles and genotyping data from 18,340 individuals across 11 countries in Asia and Europe. Further localization analyses were performed to identify genetic loci affecting the relative abundance or presence of microbial taxa, all included in the human GWAS data (Sanna et al., 2019; Kurilshikov et al., 2021). Given the complexity and the subtle genetic influences on gut microbiota, standard genome-wide significance levels (P<5×10^-8) identified through GWAS are challenging to achieve. Sample sizes in gut microbiota studies are generally smaller than those in conventional GWAS. This is largely due to the costs and complexities associated with high-throughput sequencing of the gut microbiota, which usually limit the sample size. A smaller sample size means lower statistical power, making it even more challenging to reach such stringent P-value thresholds. To capture a broader spectrum of significant associations and enhance the power of our MR analysis, we opted to utilize exposure data with a less stringent threshold of P<1×10^-5 to select our instrumental variables (Li et al., 2023). This decision was based on balancing the statistical stringency and the practical necessity of identifying a sufficient number of instrumental variables for a robust MR analysis. We aimed to minimize false discoveries while maximizing the opportunity to uncover meaningful genetic associations with gut microbiota. Furthermore, to mitigate the risk of weak IV bias, which can compromise the validity of MR analysis, we adopted a rigorous selection criterion by excluding SNP loci with F-statistics ≤10 (Burgess and Thompson, 2011). This ensures the utilization of only those SNPs with sufficiently strong associations with the exposure, thereby enhancing the reliability of our causal inferences. This approach underlines our commitment to methodological rigor and ensures the robustness of our analysis without compromising transparency.

PhenoScanner V2 (http://www.phenoscanner.medschl.cam.ac.uk/) and GWAS Catalog (https://www.ebi.ac.uk/gwas/) were utilized to further assess whether the IVs might be associated with confounding factors or risk factors for OM, thereby preventing potential pleiotropic effects (Staley et al., 2016). If the IVs were found to be related to confounders or risk factors for OM, such as infections, trauma, mental disorders, etc., they were excluded from the analysis (**Supplementary Table 3**).

#### 2.2.2 Outcome GWAS: osteomyelitis

OM summary statistics were obtained from the MRC Institute of Genetics and Molecular Medicine’s IEU Open GWAS database (https://gwas.mrcieu.ac.uk/), a repository for publicly available GWAS summary data. Considering factors like sample size, sequencing depth, ethnicity, and data update timing, we selected the dataset equivalent to the comprehensive genomic OM genetic dataset published in 2021. This dataset includes 486,484 European subjects (4,836 cases and 481,648 controls) (Sudlow et al., 2015).

#### 2.2.3 Mendelian randomization analysis

For MR analysis, we primarily used inverse variance weighting to determine the causal relationship between human gut microbiota and OM (P<0.05) (Bowden et al., 2017). We also employed three additional methods—MR-Egger (Bowden et al., 2015), weighted median (Bowden et al., 2016), and weighted mode (Hartwig et al., 2017)—for supplementary analyses. The IVW method is the cornerstone of all MR analyses, leveraging each instrumental variable’s effect estimate and its variance to assign weights. This approach emphasizes variables with smaller variances, ensuring that their impact is proportionately greater. In conjunction with IVW, both the weighted median and the weighted model serve as complementary analyses, bolstering the robustness of the findings as long as their ORs align with the IVW’s directionality. MR-Egger, distinguished by its dual utility, not only assesses heterogeneity through its P-value but also incorporates an intercept term. This term serves as a potent instrument for detecting pleiotropy, thereby enhancing the inferential strength of causal relationships in MR studies. A range of sensitivity analyses was conducted, including Cochran’s Q test (Burgess et al., 2013), funnel plots, leave-one-out analyses, and MR-Egger intercept tests. In this analysis, heterogeneity among the instrumental variables was evaluated using Cochran’s Q test, when P>0.05 indicates no heterogeneity and vice versa. Meanwhile, the potential for horizontal pleiotropy was assessed through the MR-Egger regression intercept. A P-value greater than 0.05 for the MR-Egger intercept suggests that each SNP conforms to the MR assumptions, indicating that the results derived from the IVW) method are reliable. Conversely, a P-value less than 0.05 for the MR-Egger intercept points towards the presence of potential pleiotropic biases that could influence the directionality of the causal estimates. Additionally, MR multivariate residual analysis was employed to identify and adjust for the influence of outliers among the instrumental variables, ensuring that the causal estimates are not disproportionately affected by any single SNP. The robustness of these findings was further corroborated by the leave-one-out sensitivity analysis (Hemani et al., 2017). The study’s procedure is illustrated in **Figure 1B**.

## 3 Results

**Figure 2:**
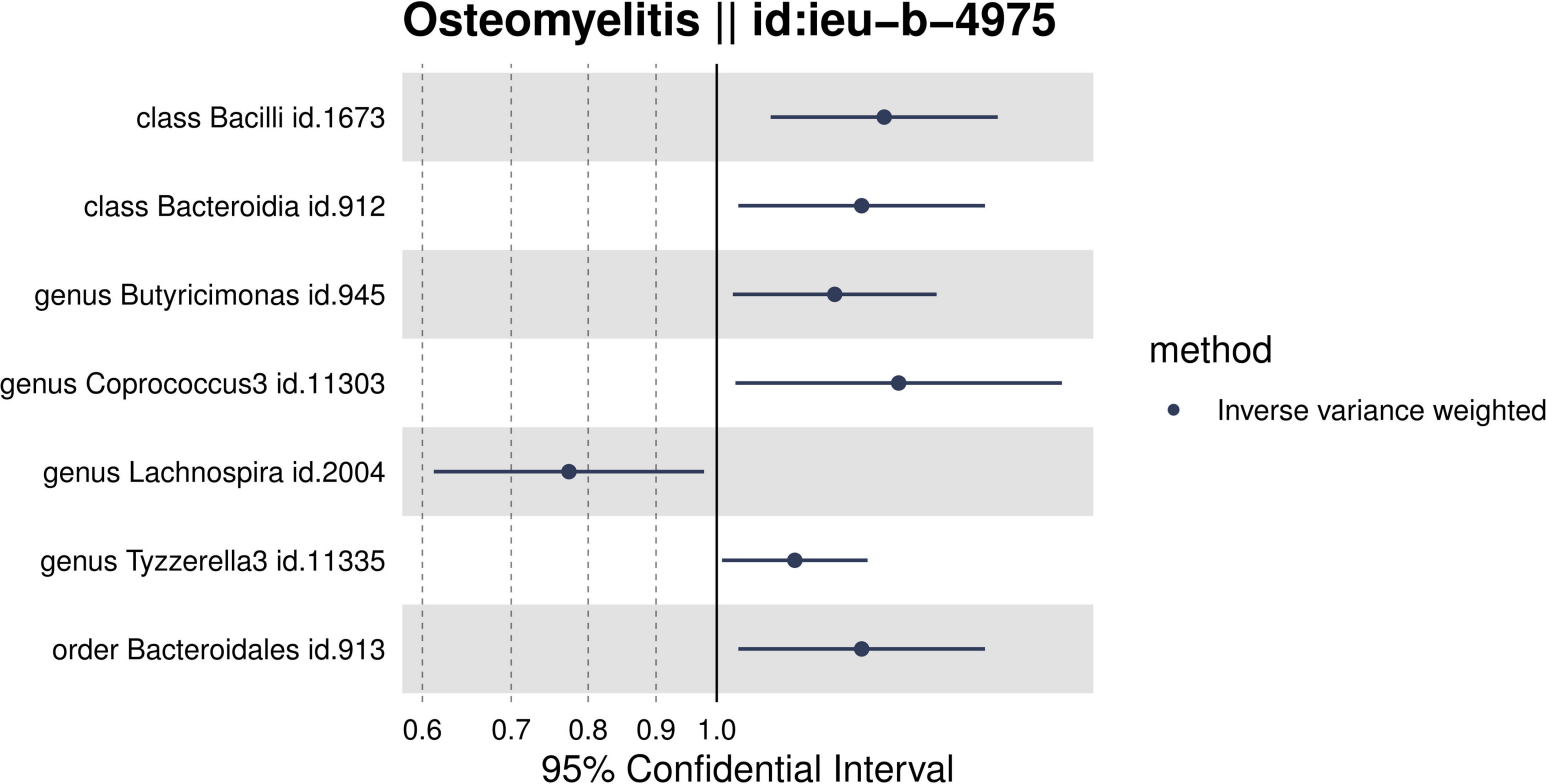
Forest plot of the associations between genetically determined 7 gut Microbiota with the risks of OM.

Through MR analysis, we identified seven intestinal microbiota with a reliable causal association with OM, as detailed in **Table 1** and depicted in a forest plot in **Figure 2**. To ensure the validity of our instrumental variables for MR analysis, SNP loci associated with these microbiota were selected based on a refined threshold of P<1×10^-5, coupled with a rigorous assessment of their instrumental strength. We excluded any SNP loci with F-statistics ≤10. The F-statistics for all utilized instrumental variables ranged from 14.82 to 35.42, confirming their adequacy as strong instruments in our study. **Supplementary Table 1** provides the F-statistics for all valid SNPs, underscoring our methodological rigor. Classified by taxonomy—kingdom, phylum, order, family, genus, species—the analysis produced significant P-value results for two orders: Bacilli (OR = 1.34, 95% CI, 1.10 to 1.63, P = 0.04) and Bacteroidia (OR = 1.29, 95% CI 1.04 to 1.59, P = 0.021); one family, specifically Bacteroidales (OR=1.29, 95% CI 1.04 to 1.59, P=0.021); and four genera: *Butyricimonas* (OR=1.23, 95% CI, 1.03 to 1.46, P=0.021), *Coprococcus3* (OR=1.37, 95% CI 1.03 to 1.82, P=0.023), *Lachnospira* (OR=0.77, 95% CI, 0.61 to 0.98, P=0.032), and *Tyzzerella3* (OR=1.15, 95% CI, 1.01 to 1.30, P=0.036). This data indicates a significant overlap of effect values at higher taxonomic levels such as phyla and orders. Our results show that *Lachnospira* may act as a protective factor against OM development. Conversely, the other identified microbiota groups—Bacilli, Bacteroidia, Bacteroidales, *Butyricimonas*, *Coprococcus3*, and *Tyzzerella3*—are positively correlated with OM incidence, suggesting they may be predisposing factors.

**Table 1:** MR results of causal links between gut microbiota and osteomyelitis risk (P < 1×10-5).

Classification		Nsnp	SE	P-value	OR (95% CI)	Pleiotropy			Heterogeneity	
						Egger intercept	SE	P- value	Q	P-value
UKB										
Class	Bacilli	16	0.101	0.004	1.34(1.10 to 1.63)	0.03	0.019	0.141	15.8	0.399
	Bacteroidia	13	0.109	0.021	1.29(1.04 to 1.59)	0.042	0.018	0.044	8.19	0.77
Order	Bacteroidales	13	0.109	0.021	1.29(1.04 to 1.59)	0.042	0.018	0.044	8.19	0.77
Genus	*Butyricimonas*	13	0.090	0.023	1.23(1.03 to 1.46)	-0.006	0.027	0.839	11.4	0.494
	*Coprococcus3*	7	0.145	0.029	1.37(1.03 to 1.82)	0.006	0.032	0.853	3.09	0.797
	*Lachnospira*	15	0.120	0.032	0.77(0.61 to 0.98)	-0.039	0.035	0.281	10.75	0.706
	*Tyzzerella3*	12	0.064	0.036	1.15(1.01 to 1.30)	-0.023	0.05	0.651	8.97	0.625

**Figure 3:**
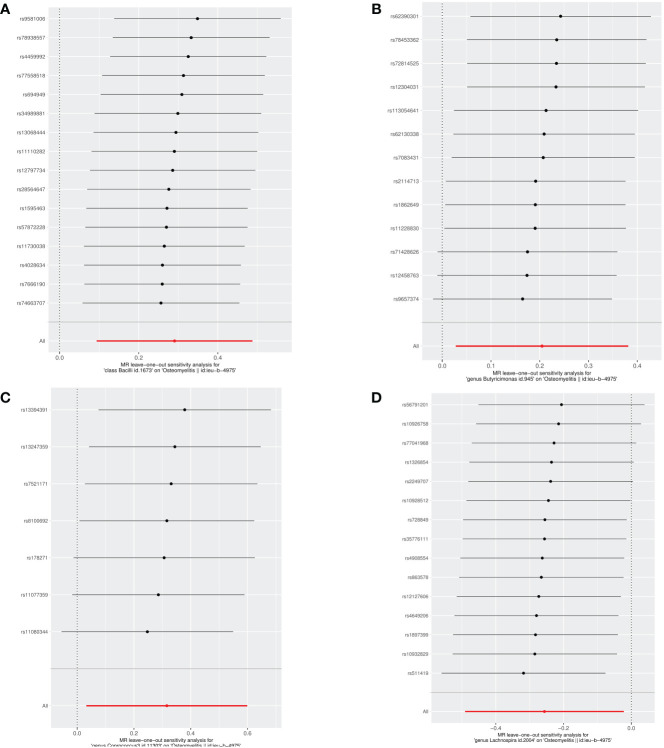
Leave-one-out analysis of the causal effects of the significant microbial taxa on OM **(A)** Causal effect of class Bacilli on OM **(B)** Causal effect of genus *Butyricimonas* on OM **(C)** Causal effect of genus *Coprococcus3* on OM **(D)** Causal effect of genus *Lachnospira* on OM.

To ensure the robustness of our study findings, we conducted several sensitivity analyses. These analyses aimed to assess potential biases and ascertain the reliability of our MR results. Initially, to evaluate the potential impact of horizontal pleiotropy on our findings, we utilized the MR-Egger intercept. Horizontal pleiotropy occurs when a gene influences multiple traits. The MR-Egger intercept can detect such bias if it exists. Our analysis showed no evidence of pleiotropic bias, as the MR-Egger intercept for each microbiome was non-zero, but all P-values were greater than 0.05, indicating that our MR study results are not affected by horizontal pleiotropy. Furthermore, we assessed the directional consistency of effect sizes using both the weighted median and weighted mode methods. These methods can provide robust estimates of causal effects, even in scenarios that might violate the instrumental variable assumptions. The results from these analyses were directly consistent with the IVW results, and the consistency across different analysis methods strengthens the validity of our findings, as summarized in **Table 2**. We also performed a leave-one-out sensitivity analysis to examine the impact of individual SNPs on our overall MR estimate. This analysis involves removing one SNP at a time and recalculating the MR estimate to check for significant outliers that could distort the analysis results. Our leave-one-out test did not identify any outliers, further confirming the stability and robustness of our study results against individual SNP variations. **Figures 3A–D** showcase the leave-one-out sensitivity analysis results for the significant microbial taxa, specifically class Bacilli, genus *Butyricimonas*, genus *Coprococcus3*, and genus *Lachnospira*, respectively. Through this analysis, we did not identify any single SNP that would significantly alter the overall effect for any of the studied microbial taxa. This indicates the robustness of our findings, underscoring the stability of the causal relationships identified between these microbial taxa and OM Lastly, to assess heterogeneity within our data, which could indicate varying effects of SNPs on the outcome, we applied Cochran’s Q test. A lack of significant heterogeneity was confirmed, with all P-values exceeding 0.05. This suggests that the instrumental variables used in our analysis exert uniform effects, enhancing the reliability of our MR study findings.

**Table 2:** Analysis of the results of 4 MR methods between gut microbiota and OM.

Level	Microbiota	SNPs	Methods	Beta	OR (95% CI)	*p* value
Class	Bacilli	16	Inverse variance weighted	0.29	1.34 (1.10,1.63)	0.00
			MR Egger	-0.08	0.93 (0.56,1.53)	0.77
			Weighted median	0.20	1.23 (0.92,1.63)	0.16
			Weighted mode	0.06	1.06 (0.68,1.67)	0.80
	Bacteroidi	13	Inverse variance weighted	0.25	1.29 (1.04,1.59)	0.02
			MR Egger	-0.22	0.80 (0.51,1.27)	0.37
			Weighted median	0.21	1.23 (0.91,1.66)	0.18
			Weighted mode	0.24	1.27 (0.86,1.88)	0.25
order	Bacteroidales	16	Inverse variance weighted	0.25	1.29 (1.04, 1.59)	0.02
			MR Egger	-0.22	0.80 (0.51, 1.27)	0.37
			Weighted median	0.21	1.23 (0.92, 1.64)	0.16
			Weighted mode	0.24	1.27 (0.86, 1.88)	0.25
Genus	*Butyricimonas*	13	Inverse variance weighted	0.20	1.23 (1.03, 1.46)	0.02
			MR Egger	0.27	1.30 (0.71, 2.38)	0.41
			Weighted median	0.17	1.18 (0.92, 1.52)	0.19
			Weighted mode	0.29	1.33 (0.84, 2.11)	0.24
	*Coprococcus3*	7	Inverse variance weighted	0.32	1.37 (1.03, 1.82)	0.03
			MR Egger	0.22	1.25 (0.47, 3.33)	0.68
			Weighted median	0.32	1.38 (0.95, 1.99)	0.09
			Weighted mode	0.29	1.34 (0.79, 2.28)	0.32
	*Lachnospira*	15	Inverse variance weighted	-0.26	0.77 (0.61, 0.98)	0.03
			MR Egger	0.48	1.62 (0.44, 5.95)	0.48
			Weighted median	-0.26	0.77 (0.56, 1.07)	0.12
			Weighted mode	-0.13	0.88 (0.48, 1.60)	0.68
	*Tyzzerella3*	12	Inverse variance weighted	0.14	1.15 (1.01, 1.30)	0.04
			MR Egger	0.29	1.34 (0.68, 2.66)	0.42
			Weighted median	0.1	1.10 (0.93, 1.31)	0.28
			Weighted mode	0.08	1.09 (0.82, 1.45)	0.57

**Figure 4:**
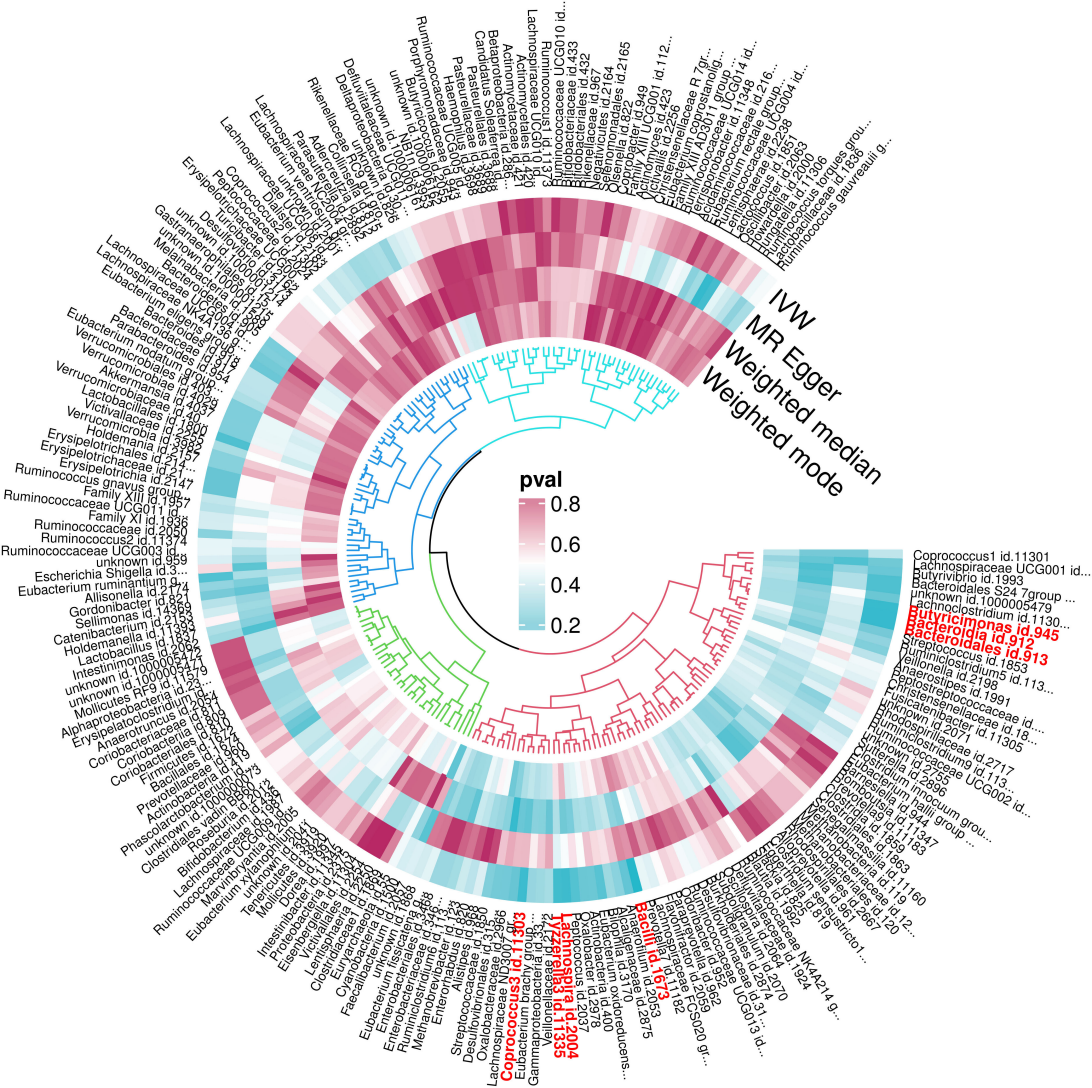
P-value results of MR of 211 gut microbiota and OM.

For a comprehensive overview, **Figure 4** details the MR findings for all 211 gut microbiota associated with OM, with the key taxa highlighted in red indicating significant associations (IVW results, P < 0.05). For extensive insights into all analyzed microbiota, please refer to **Supplementary Table 2**.

## 4 Discussion

In this MR study, we initially investigated potential causal links between gut microbiota and OM risk by leveraging large-scale summary statistics from gut microbiota and OM GWAS. Notably, seven bacterial traits were identified as having a significant causal association with OM risk.

At first glance, there appears to be no connection between the gut microbiota and OM. However, a new perspective was introduced by Zhao et al. in their 2021 study (Zhao et al., 2021). Their research revealed that prolonged antibiotic treatment for OM disrupts the gut microbiota. This disruption significantly interferes with bone consolidation by elevating pro-inflammatory cytokine levels. Additionally, an earlier article published in JAMA also discussed the occurrence of localized osteomyelitis caused by Group B Streptococcus in the absence of any other infection sources. It was found that healthy adults can be carriers of Group B Streptococcus in their gut microbiota, suggesting the gut as a potential origin for clinical infections (McGuire et al., 1977). Based on these clinical evidences, our study delves deeper into the complex interplay between the gut microbiome and OM. We identified three primary pathways through which gut microbiota impacts distant organs: the modulation of nutrient absorption, alteration of the immune system in intestinal endothelial cells, and translocation of gut microbiota or their metabolites across the endothelial barrier into systemic circulation. Research shows that diet significantly affects the composition and abundance of intestinal microbiota in mice. Studies examining inflammation and immunity responses in mice with varied diets suggest that intestinal microbiota adaptively modulates its biological functions. This concept was further supported by a study from Phillips et al., 2022 (Rausch et al., 2022), which found that mice with OM fed a high-fat diet had a higher proportion of Lactobacillus and Prevotella in their gut microbiome compared to those on a standard diet, who also developed OM. Notably, mice on the high-fat diet had lower IL-1b levels in their pedis. Crucially, fecal microbiota transplantation from high-fat diet mice to standard diet mice before disease onset not only inhibited Prevotella growth but also reduced IL-1b synthesis, thus preventing OM. This finding highlights the potential of diet-induced changes in gut microbiota to affect the initiation and progression of OM (Phillips et al., 2016). In the human gastrointestinal tract, the intestinal epithelial barrier serves as a key defense against harmful antigens and pathogens and houses the gut microbiota. This barrier is vital for nutrient absorption and immune defense. Normally, tight junction proteins seal the intestinal epithelium securely, offering protection. However, during intestinal dysbiosis, gut microbiota can disrupt the expression and localization of TJ proteins, thereby increasing intestinal permeability and leading to extensive inflammatory reactions, including severe conditions like OM (Tu et al., 2021). Bacterial populations exert a significant impact on the production of pro-inflammatory cytokines through intricate interactions with the host’s immune system. Key bacterial constituents, such as lipopolysaccharides and peptidoglycans, are recognized as powerful immunomodulators (Hancock et al., 2012). They are capable of triggering the release of pro-inflammatory cytokines by activating the host’s pattern-recognition receptors, notably Toll-like receptors. The cytokines released, including tumor necrosis factor α, interleukin 1β, and interleukin 6, are pivotal in orchestrating the inflammatory response and facilitating bone remodeling processes. Within the context of osteomyelitis, bacterial infections—whether situated directly within the bone/bone marrow or disseminated to these locales via the bloodstream—act to augment the production of these pro-inflammatory cytokines. This augmentation, in turn, disrupts the bone consolidation process (Kubatzky et al., 2018). The disruption involves not only the activities of native cellular types, such as osteoblasts and chondrocytes, but also extends to immune cells, including macrophages and neutrophils. These immune cells migrate to the infection site amidst the inflammatory process, thereby participating actively in the ensuing inflammatory response. Furthermore, studies on the gut-bone axis have shown that short-chain fatty acids, metabolites of gut microbiota, play a crucial role in promoting regulatory T cells development, inhibiting Th17 cell production, reducing inflammatory cytokine synthesis, and maintaining systemic immune balance (Yan et al., 2016).

In the broader context of our study on the gut-bone axis and OM, it’s imperative to consider the interplay between mental health and gut microbiota. As highlighted in a pivotal 2021 study by Gulistan Agirman et al., published in Science, the crosstalk within the gut-brain axis can significantly modulate inflammatory responses and immune homeostasis, impacting the whole organism (Agirman et al., 2021). This underscores the potential of gut microbiota, through various pathways such as the autonomic nervous system, neurotransmitters, and the immune system, to interact with mental health disorders including depression, anxiety, and autism. Furthermore, research conducted by Chen et al., 2023 revealed a significant increase in the risk of OM among patients with major depressive disorder (Odds Ratio=1.44, 95% Confidence Interval: 1.18~1.874) (Chen et al., 2023), underscoring the necessity to consider mental health’s potential impact when analyzing the relationship between gut microbiota and OM.Therefore, our research into the correlation between gut microbiota and OM must account for the influence of mental health status on the composition and functionality of gut microbiota, and how these alterations might affect the development and progression of OM.

“Bacilli, a category of Gram-positive bacteria, are notable for their production of resistant endospores, broad-spectrum bacillary activity, and synthesis of bacteriocins. They are commonly found in various biological environments and form a significant part of the human intestinal microbiota. Although most Bacilli are non-invasive, there are exceptions, with *Bacillus cereus* being a notable example. According to Veysseyre et al., 2018, *Bacillus cereus* was implicated in 57 infections, of which 10 involved bones and joints. This observation suggests that such infections might occur when intestinal microbiota or their metabolic byproducts cross the endothelial barrier, enter the bloodstream, trigger an inflammatory response, and potentially lead to a systemic inflammatory reaction (Veysseyre et al., 2015). In a related study, Bradley et al., 2014 found that *Bacillus cereus* can compromise the human intestinal barrier through a binary protein mechanism. This mechanism involves the bacteria and their toxins disrupting the human intestinal barrier by achieving receptor-mediated endocytosis, using the AB toxin complex for cellular intoxication, which comprises ADP-ribosyltransferase (A) and cell-binding (B) components. This discovery supports the hypothesis that such disruptions could be a contributing factor in the pathogenesis of OM (Stiles et al., 2014), which is consistent with the conclusions we have drawn from our study.

Our findings indicate that both Bacteroidia and Bacteroidales are positively associated with an increased risk of developing OM. Bacteroidales, belonging to Gram-negative bacteria, are a crucial component of the intestinal microbiota and possess one of the most intricate polysaccharide-pod systems in bacteria, featuring at least eight different polysaccharides (PSA-PSH). On another front, the lipopolysaccharides of the genus Anabaena, which lack O antigens, demonstrate a virulence approximately 1000 times greater than E. coli lipopolysaccharides. In contrast, the fragile anaplasma toxin, a key virulence factor in the *Anaplasma genus*, exists in three isoforms (BFT-1, BFT-2, BFT-3), with BFT-2 identified as the primary agent of tissue damage. This highly potent toxin, BFT-2, is capable of crossing the intestinal epithelial barrier, entering the bloodstream, and potentially initiating hematogenous OM. Additionally, the ability to withstand oxidative stress is a significant contributor to pathogenic virulence. Bacteria regulate the production and transport of various oxidoreductases, such as catalase, peroxidase, and thioredoxin, under the control of the transcription factor OxyR. This regulation enhances bacterial resilience against the host’s oxidative responses, thus promoting increased tolerance (Zafar and Saier, 2021). Research conducted by Zhao et al., 2021 has established that the prolonged use of antibiotics in OM treatment results in a reduction of the Bacteroidetes population, leading to decreased levels of inflammation (Zhao et al., 2021). This evidence indirectly corroborates our hypothesis that Bacteroidia or Bacteroidales play a role in the development and progression of OM, mirroring the conclusions we reached through our MR analysis.

In a notable case study, Panagiota et al., 2022 reported two cases of vertebral OM caused by *Bacillus fragilis*, with no detectable infection foci elsewhere in the body (Rendoumi et al., 2022). This finding supports the hypothesis of a potential link with enterobacteria, offering valuable insights for future research (Zafar and Saier, 2021). Gut microbiota dysbiosis, characterized by an imbalance in microbial populations leading to the loss of vital physiological functions, disrupts the intestinal microbial ecosystem. This alteration in gut microbiota composition is thought to significantly affect the development and manifestation of OM. It primarily does so by influencing the production of short-chain fatty acids (SCFAs), altering intestinal permeability, and modulating immune and inflammatory responses. Among SCFAs, butyrate is a key component, predominantly produced through the enzymatic breakdown of dietary fibers in the intestine and serving as a major energy source for intestinal epithelial cells (Lee, 2022). Notable butyrate-producing bacteria include rumen *Coccidioides*, *Vibrio butyronucleicus*, and *Aeromonas butyricola*. Butyrate functions diversely by interacting with G protein-coupled receptors and participating in local and extensive signaling networks. These interactions contribute to strengthening intestinal barrier function, mucosal immunity, and maintaining intestinal homeostasis, potentially enhancing energy metabolism, aiding weight loss, reducing inflammation, and balancing the gut-brain axis. However, a decrease or shift in the population of butyrate-producing bacteria like Aeromonas butyric acidophilus can weaken the body’s defensive mechanisms, leading to an imbalanced immune system and increased inflammation.

Conversely, *Lachnospira*, identified in our study as the sole genus potentially acting as a protective agent, belongs to the phylum of Thick-walled Bacteria. It is believed that *Trichoderma*, a bacterium likely to be beneficial, participates in the metabolism of various carbohydrates. This process results in the production of crucial substances like acetic acid and butyric acid, which provide energy to the host (Knip and Siljander, 2016; Carasso et al., 2021). Predominantly found in early infant populations, the prevalence of *Lachnospira* has been associated with increased levels of short-chain fatty acids. These SCFAs are vital, particularly in maintaining the integrity of the intestinal barrier and exhibiting anti-inflammatory properties. Not only do they ensure efficient nutrient absorption, but they also protect against the entry of harmful substances (such as bacterial endotoxins) into the systemic circulation, thereby reducing the risk of hematogenous OM (Parada Venegas et al., 2019). Previous literature on the association of *Lachnospira* with OM or inflammation is scarce, paving the way for future research in this area.

It is noteworthy that dysbiosis of the osseous microbiota could lead to disturbances in the host’s microbiome, thereby contributing to the development of OM (Chen et al., 2021). For instance, a diverse microbial community within bone tissues may serve as a defense against pathogen invasion, whereas an imbalance in these microbial communities could facilitate the proliferation of pathogens, culminating in OM. Additionally, the microbiota associated with bone tissues may play a crucial role in modulating local immune responses. An imbalance can weaken immune defenses, rendering bones more susceptible to infection (Weitzmann, 2017). In certain scenarios, bacteria might even form hard-to-eradicate biofilms on bone surfaces, leading to chronic infections. Moreover, the interaction between osseous microbiota and non-osseous microbiota, such as the gut microbiota, could indirectly impact bone health through systemic inflammation and immune responses. These insights underscore the importance of not only considering gut microbiota but also focusing on the role and imbalance of bone microbiota in the development of OM. It should be noted that current research on the relationship between bone microbiota and OM remains relatively limited. Present studies typically focus on identifying specific microbes present in diseased versus healthy tissues and understanding how these microbes interact with the host’s immune system. To comprehensively understand the relationship between dysbiosis of bone microbiota and OM, future research will need to employ advanced sequencing and analytical techniques (Lagier et al., 2015).

The innovative approach of this study establishes the foundation for the first MR analysis to determine a causal relationship between gut microbiota and OM. By eliminating confounding factors, this research highlights candidate bacteria for future functional studies. Moreover, the implications of these MR findings are significant in public health, enriching previous research on the gut microbiota-OM connection by introducing a genetic perspective. From a preventive medicine standpoint, these insights could inform strategies for OM prevention through timely gut microbiota modulation. Diagnostically, they emphasize the importance of vigilant OM screening and early detection in individuals with gut microbiota irregularities.

## 5 Conclusion

In conclusion, this study marks the first instance where compelling evidence has been presented demonstrating the significant impact of gut microbiota on OM. By identifying several key bacterial groups, including Bacilli, Bacteroidia, Bacteroidales, *Butyricimonas*, *Coprococcus3*, *Tyzzerella3*, and *Lachnospira*, our research suggests these taxa as potential novel biomarkers. These critical findings herald a new phase in developing treatment and prevention strategies for OM. For example, when osteomyelitis is suspected at an early stage in the clinical setting, feces from patients can be tested to see if the abundance of these genera is elevated compared to normal human samples, so that early diagnosis and treatment can be made in advance. Secondly, it is possible to create inhibitors of certain genera such as Bacteroidia, and Bacteroidales in our conclusions to intervene or treat OM, or to make *Lachnospira*-related probiotics to assist in the treatment of OM. However, it remains essential to conduct further studies. These future investigations should aim to clarify the precise relationship between gut microbiota and OM and to fully understand the specific mechanisms underpinning this association. Only then can they truly provide an effective approach to the treatment and prevention of osteomyelitis. This will in turn benefit many people with osteomyelitis and reduce the burden on society!

## Data availability statement

Publicly available datasets were analyzed for this study. These data can be found here: Gut microbiota GWASD data from MiBioGen (https://mibiogen.gcc.rug.nl/). Osteomyelitis GWAS data from the UK Biobank (https://gwas.mrcieu.ac.uk/datasets/ieu-b-4975/).

## Ethics statement

The manuscript presents research on animals that do not require ethical approval for their study.

## Author contributions

WZ: Methodology, Writing – original draft, Writing – review & editing. YW: Conceptualization, Writing – original draft. XL: Writing – review & editing, Formal analysis. DC: Writing – original draft. LM: Software, Writing – original draft. JZ: Writing – review & editing. FH: Writing – original draft. ZJ: Writing – original draft, Writing – review & editing.
